# Prevention of airway inflammation with topical cream containing imiquimod and small interfering RNA for natriuretic peptide receptor

**DOI:** 10.1186/1479-0556-6-7

**Published:** 2008-02-15

**Authors:** Xiaoqin Wang, Weidong Xu, Subhra Mohapatra, Xiaoyuan Kong, Xu Li, Richard F Lockey, Shyam S Mohapatra

**Affiliations:** 1 Clinical Laboratory Center of First Affiliated Hospital, Xi'an Jiaotong University College of Medicine, Xi'an, China; 2 Division of Allergy and Immunology, Culverhouse Airway Disease and Nanomedicine Research Center, University of South Florida College of Medicine, Tampa, Florida, USA; 3 Endocrinology Division, Internal Medicine, University of South Florida College of Medicine, Tampa, Florida, USA; 4 James A. Haley VA Medical Center, Tampa, Florida, USA

## Abstract

**Background:**

Asthma is a complex disease, characterized by reversible airway obstruction, hyperresponsiveness and chronic inflammation. Principle pharmacologic treatments for asthma include bronchodilating beta2-agonists and anti-inflammatory glucocorticosteroids; but these agents do not target the main cause of the disease, the generation of pathogenic Th2 cells. We previously reported reduction in allergic inflammation in mice deficient in the ANP receptor NPRA. Here we determined whether siRNA for natriuretic peptide receptor A (siNPRA) protected against asthma when administered transdermally.

**Methods:**

Imiquimod cream mixed with chitosan nanoparticles containing either siRNA green indicator (siGLO) or siNPRA was applied to the skin of mice. Delivery of siGLO was confirmed by fluorescence microscopy. The anti-inflammatory activity of transdermal siNPRA was tested in OVA-sensitized mice by measuring airway hyperresponsiveness, eosinophilia, lung histopathology and pro-inflammatory cytokines.

**Results:**

SiGLO appearing in the lung proved the feasibility of transdermal delivery. In a mouse asthma model, BALB/c mice treated with imiquimod cream containing siNPRA chitosan nanoparticles showed significantly reduced airway hyperresponsiveness, eosinophilia, lung histopathology and pro-inflammatory cytokines IL-4 and IL-5 in lung homogenates compared to controls.

**Conclusion:**

These results demonstrate that topical cream containing imiquimod and siNPRA nanoparticles exerts an anti-inflammatory effect and may provide a new and simple therapy for asthma.

## Introduction

Chitosan is a natural cationic polysaccharide extracted from crustacean shells. It is a good candidate for the delivery of genes and drugs because of its biodegradability, biocompatibility, mucoadhesiveness, low immunogenicity, and strong immunostimulatory properties [1-3]. It has been found to have beneficial properties including anticoagulant, wound-healing and anti-microbial activities. Chitosan has also been widely used in controlled drug delivery [4-7] because it is nontoxic, nonhemolytic, slowly biodegradable and capable of encapsulating a drug or DNA to protect it from enzymatic degradation. The interaction between cationic amino groups on chitosan and anionic moieties such as sulfonic acid on the mucus layer enhances its muco-adhesiveness. Furthermore, chitosan is known to cross the epithelial barrier through tight junctions [8]. We have reported on chitosan delivery of vector-driven small interfering RNA (siRNA) intranasally to protect mice from respiratory syncytial virus infection [3].

While oral and intranasal routes of drug delivery are commonly used, each of these routes has its limitations. For example, orally delivered drugs have to undergo first-pass metabolism which can rapidly inactivate them. The nasal route may be inadequate for infants and children with congested noses due to allergy or infection. Transdermal delivery may be the ideal modality because skin is the most accessible organ of the body and the route with the highest therapeutic compliance; but for transdermal entry of DNA only liposomes and polymers have had limited success [9-11].

Since the size of the sweat pores and the follicular openings of the skin are 30 to 100 μm, it is reasonable to expect that nanocomplexes would facilitate the penetration through the skin of DNA or small oligonucleotides such as siRNAs [12]. siRNAs have become a powerful tool for gene silencing and have the potential to become the preferred form of treatment for cancer and infectious disease. The combination of gene-silencing through siRNA with the greatly enhanced delivery offered by nanoparticles provides a therapeutic system with a high degree of flexibility, specificity and safety. Previously, cationic lipids were reported to successfully deliver siRNA across mucosal surfaces [13,14]. In this report, we developed and tested a topical siRNA delivery system based on chitosan nanoparticles. The natriuretic peptide receptor A (NPRA) was selected as the siRNA target because it was recently found that NPRA knockout prevented lung inflammation in a mouse model of allergic asthma. NPRA is the primary receptor for atrial natriuretic peptide (ANP), which has been associated with allergic inflammation and asthma. NPRA is expressed on cells in many different tissues of various organ systems and the cell-surface receptor contains an intrinsic guanylyl cyclase that is activated by ANP binding. ANP signals primarily through NPRA by increasing cGMP and activating cGMP-dependent protein kinase (PKG). Activated PKG turns on ion transporters and transcription factors, which together affect cell growth and proliferation, and inflammation [15].

To test whether topical delivery of siRNA for NPRA can reduce chronic inflammation of the lung in an experimental asthma model, 5% imiquimod cream was mixed with siNPRA nanoparticles. Imiquimod cream has two advantages in our test: first, imiquimod itself has been reported to modulate airway inflammation [16,17] when given intranasally; secondly, the cream contains the penetrating agent polysorbate 60 [18] which facilitates the penetration siRNA nanoparticles through the skin. Imiquimod, as a TLR-7 agonist, was reported to have Th1-biased immune responses by increasing TNF-α and IL-12 in dendritic cells [19]. By combining the treatment of imiquimod and siNPRA nanoparticles, we anticipated that more protection against airway inflammation would be achieved in a mouse model of asthma.

## Materials and Methods

### Cell lines

The HEK293 cell line was purchased from ATCC (Rockville, MD) and the human prostate cancer cell line PC3 was kindly provided by Dr. Wenlong Bai at the University of South Florida. All three cell lines were grown in Earle's modified Eagle's medium supplemented with 10% fetal bovine serum at 37°C in a 5% CO_2 _incubator. HEK-GCA, a stable cell line overexpressing NPRA, was established in our lab. HEK-GCA was grown in Dulbecco's modified Eagle's medium with 10% fetal bovine serum and 1 μg/ml hygromycin.

### SiRNA: siGLO and siNPRAs

As siRNA marker, siGLO green indicator was purchased from Dharmacon Research Inc. For siRNA against NPRA, several targeting sequences were located using siRNA finder software (Ambion, Austin, TX). Vector-driven siNPRA were constructed by cloning the annealed siNPRA oligonucleotide primers between the *Apa *I and *EcoR *I sites of pSilencer-1.0 (Ambion, Austin, TX). The resulting siNPRA plasmids were used to transfect HEK-GCA cells. Thirty six hours after transfection, cells were harvested and cell lysates subjected to Western blot assay to determine which siNPRA construct gave the best inhibition in NPRA expression. Each siNPRA construct was also given to mice intranasally to confirm its effectiveness. The most effective siNPRA we tested has the sequence: GGGCGCUGCUGCUGCUACCdTdT (sense). The scrambled siRNA is a random rearrangement of the normal siNPRA with the sequence CGUCGAGUGCCGUCGUGCCdTdT. The synthetic siNPRA was prepared by annealing the sense siNPRA oligonucleotide strand and the antisense strand by following the instructions of Integrated DNA Technologies, Inc. (Coralville, IA).

### Animals

Female BALB/c and nude mice, 6–8 weeks of age, were purchased from Jackson Laboratory (Bar Harbor, ME). NPRA^-/- ^C57BL/6 mice were kindly provided by Dr. William Gower at the University of South Florida. All mice were maintained in a pathogen-free environment, and all procedures were reviewed and approved by the University of South Florida Institutional Committee on Animal Research. Mice were tested for siNPRA efficacy in blocking NPRA expression first, and then for protection against airway inflammation in an ovalbumin sensitization and challenge model.

### Preparation and characterization of siRNA chitosannanoparticles

Preparation and characterization of siRNA chitosan nanoparticles was performed as previously described [5]. Briefly, chitosan (33 *k*D*a*, with 90% deacetylation) was obtained from TaeHoon Bio (Korea). Chitosan stock solution (10 mg/ml) was prepared in 1% acetic acid. siGLO, siNRNA or pEGFP-N2 plasmid DNA were mixed with chitosan at a ratio of 1:5 (wgt:wgt). After chitosan was added to the diluted DNA or siRNA solution, the mixture was vortexed vigorously for 20–30 sec and stored at room temperature until use. For transfection of siGLO and pEGFP-N2, HEK293 cells were grown on 6-well plates were incubated with chitosan nanoparticles containing 200 pmol of siGLO or 1 ug of pEGFP-N2. Eight hours later the cells were washed with PBS and recultured in regular medium. However, lipofectamine 2000 (Invitrogen, CA) was used to transfect siNPRA into HEK-GCA cells to evaluate the inhibition of NPRA expression by siNPRA though Western blot assay. For topical administration of siGLO or siNPRA to the back of each mouse, 2 nmol of siGLO or 5 nmol of siNPRA were complexed with 50 μg or 125 μg of chitosan, respectively, before mixing with imiquimod cream. Intranasally delivered pEGFP-N2 was selected as a positive control for whole-body fluorescence imaging. In this assay, 25 μg of pEGFP-N2 was complexed with 125 μg of chitosan and vortexed for 20 minutes before being given to mice as nasal drops.

### Western blots

HEK-GCA cells were grown in 6-well plates and transfected with 200 pmol of siNPRA or scrambled siRNA (Scr) using Lipofectamine 2000 according to the manufacturer's instructions (Invitrogen, CA). To extract whole-cell protein, cells were harvested 48 h after transfection and resuspended in lysis buffer containing 50 mM HEPES, 150 mM NaCl, 1 mM EDTA, 1 mM EGTA, 10% glycerol, 0.5% NP-40, 0.1 mM phenylmethylsulfonyl fluoride, 2.5 μg/ml leupeptin, 0.5 mM NaF, and 0.1 mM sodium vanadate. Fifty μg of protein was subjected to sodium dodecyl sulfate-polyacrylamide gel electrophoresis on a 10% polyacrylamide gel and then transferred onto nitrocellulose membranes. Western blot assay was performed according to the manufacturer's instructions (Cell Signaling Technology, Beverly, MA).

### Modulation of lung inflammation by siNPRA

Sixteen BALB/c mice were divided into four groups (n = 4 per group). One group served as naïve control with no OVA sensitization or challenge and no siRNA nanoparticle treatment. The second group received OVA sensitization (50 μg OVA i.p. injected on day 1 and day 7) and OVA challenge (25 μg intranasally on day 18, 19, 20 and 21). Animals in the third group got OVA sensitization, Ova challenge and transdermal treatment with siNPRA nanoparticles (containing 5 nmol of siNPRA on day 18, 19, 20, and 21). The last group was OVA-sensitized and – challenged, but treated with scrambled siRNA nanoparticles (containing 5 nmol of siNPRA on day 18, 19, 20 and 21). To prepare siNPRA nanoparticles, synthetic siNPRA was complexed with chitosan by mixing 5 nmol of siNPRA with 150 μg of chitosan polymers. The chitosan and siNPRA mixture was vortexed vigorously for 30 seconds and stored at room temperature until use. SiNPRA nanoparticles were given to mice intranasally or transdermally. When given transdermally, siNPRA nanoparticles containing 5 nmol of siNPRA were mixed with 62.5 mg of 5% imiquimod cream (3 M pharmaceuticals, Northridge, CA) which contains the penetrating agent polysorbate 60 and applied to shaved skin on the backs (above the lung) of the BALB/c mice. A control group received the same amount of scrambled siRNA nanoparticles mixed with imiquimod cream. All mice were sacrificed to collect BAL fluid. Mouse lungs were rinsed with intratracheal injections of PBS then perfused with 10% neutral buffered formalin. Lungs were removed, paraffin-embedded, sectioned at 20 μm, stained with hematoxylin and eosin (H & E) and examined under the microscope to determine lung pathology.

### Differential cell enumeration in bronchoalveolar lavagefluid

Bronchoalveolar lavage (BAL) fluid was collected and differential cell counts were performed as previously described [7]. Briefly, BAL was centrifuged and the cell pellet was suspended in 200 μl of PBS and counted using a hemocytometer. The cell suspensions were then centrifuged onto glass slides using a cytospin centrifuge at 1000 rpm for 5 min at room temperature. Cytocentrifuged cells were air dried and stained with a modified Wright's stain (Leukostat, Fisher Scientific, Atlanta, GA) which allows differential counting of monocytes and lymphocytes. At least 300 cells per sample were counted by direct microscopic observation.

### Determination of airway hyperreactivity (AHR)

AHR, expressed as enhanced pause (Penh), was measured in unrestrained mice by whole body plethysmography (Buxco, Troy, NY). Groups of mice (n = 4) were exposed for 5 min to nebulized PBS to establish a baseline then to increasing concentrations (6–25 mg/ml) of nebulized methacholine (MCh; Sigma, St. Louis, MO) in PBS. Challenges were done for 5 min followed by recordings of Penh for 5 min. The Penh values were averaged and expressed for each MCh concentration as a percentage of the baseline reading.

### Statistics

A minimum of four mice was used in each test group. Experiments were repeated at least once and measurements were expressed as means plus or minus standard error of the mean or standard deviation. Comparisons of groups were done using a two-tailed Student's *t *test and p < 0.05 was considered significant.

## Results

### Transdermal delivery of siGLO using chitosan nanoparticles

**Figure 1:**
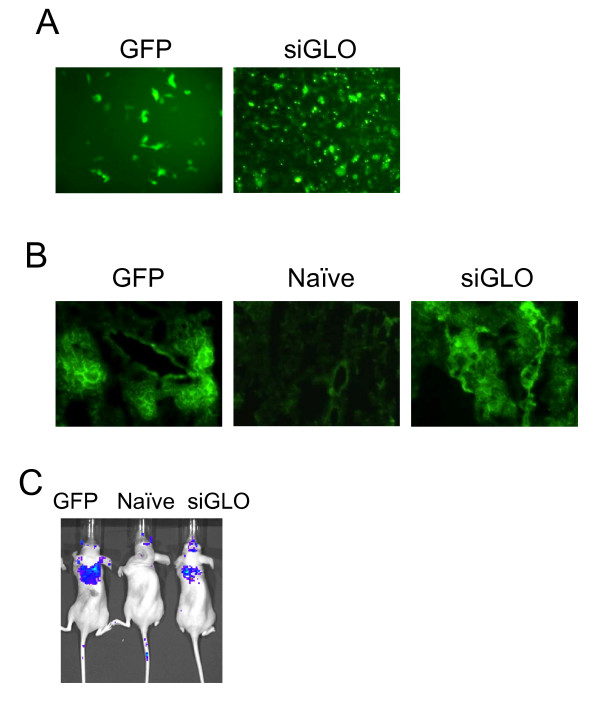
**Delivery of siGLO chitosan nanoparticles *in vitro *and *in vivo***. (**A**) HEK293 cells were transfected with 200 pmol of siGLO complexed with 5 μg of chitosan nanoparticles. Fluorescent cells containing siGLO were observed by fluorescence microscopy. HEK293 cells were also transfected with chitosan nanoparticles containing green fluorescent protein expression plasmid, pEGFP-N2, as a positive control. (**B**) The green fluorescence from the frozen lung sections of mice treated transdermally with siGLO or intranasal pEGFP-N2 nanoparticles was monitored by fluorescence microscopy. (**C**) siGLO nanoparticle cream containing 2 nmol of siGLO was spread on the backs of BALB/c nude mice, and a second dose of siGLO nanoparticles was administered 24 h later. The transdermally-delivered siGLO was detected 48 h after the initial treatment by *in vivo *imaging using the Xenogen IVIS system. Mice receiving intranasal pEGFP-N2 chitosan nanoparticles were included as positive control for *in vivo *imaging.

First, we tested if chitosan polymers can help to transfect cells with siRNA *in vitro *using siGLO as a fluorescent siRNA marker [20]. To prepare siGLO-chitosan nanoparticles, 0.2 nmol of siGLO were complexed with 5 mg of chitosan polymers (33 *k*D*a*) before transfection. HEK293 cells were transfected and the incorporation of siGLO into HEK293 cells was monitored by fluorescence microscopy 24–48 hrs after transfection (Fig. 1A). HEK293 cells were also transfected with pEGFP-N2 chitosan nanoparticles as a positive control (Fig. 1A). Next, lung sections were prepared from siGLO-treated mice and the presence of siGLO in the lung was confirmed by fluorescence microscopy (Fig. 1B). We also tested if chitosan nanoparticles could deliver siGLO transdermally in mice. SiGLO chitosan nanoparticles (2 nmol siGLO plus 50 mg of chitosan) were mixed with 62.5 mg of 5% imiquimod cream and applied to the backs of BALB/c nude mice. A second application was done at the same location 24 hrs later. Distribution of siGLO *in vivo *was detected through whole-body fluorescence imaging using a Xenogen IVIS system. SiGLO was found to reach the lung 48 hrs after treatment (Fig. 1C). Intranasally-delivered pEGFP-N2 nanoparticles (without cream) were included as a positive control for the presence of fluorescence (Fig. 1A–C).

### NPRA deficiency reduced lung inflammation in a mouse asthma model

**Figure 2:**
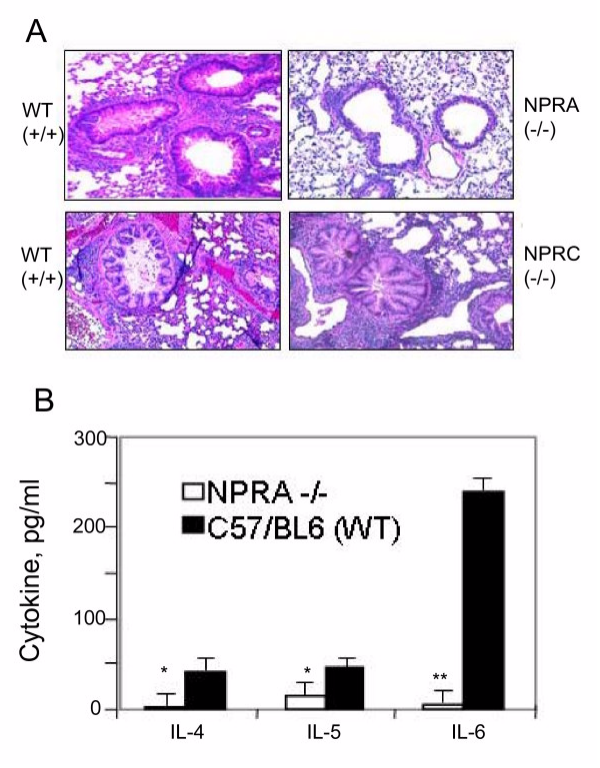
**NPRA knockout prevents allergic airway inflammation**. (**A**) Knockout of NPRA but not NPRC attenuates airway inflammation. C57BL6 wild type, NPRA-/- and NPRC-/- knockout mice were OVA-sensitized (i.p.) at day zero and day seven and challenged twice with OVA (i.n.). Two days later, mice were sacrificed and lung sections were stained with hematoxylin/eosin. (**B**) BAL fluids were obtained from WT and NPRA-/- mice and assayed by ELISA for pro-inflammatory cytokines, IL-4, -5 and -6. Results shown are averages of two separate experiments with standard deviations (*, *P *< 0.05, **, *P *< 0.01).

Current pharmacologic treatments for asthma act only on symptoms and do not target the main cause of the disease, the generation of pathogenic Th2 cells [21-23]. Hence, there is a continued search for new therapeutic agents against allergy and asthma. Since plasma ANP levels have been shown to increase during asthma exacerbation [18], we used mice deficient in the receptor for ANP (NPRA^-/-^) to examine the role of the ANP pathway in lung inflammation and asthma. In the mouse model of asthma, C57BL/6 wild type, NPRA-/- and NPRC-/- knockout mice were sensitized intraperitoneally (i.p.) with ovalbumin (OVA), the allergen used in the mouse model of allergic asthma, and then challenged with OVA intranasally (i.n.). NPRA-/- mice mounted little inflammatory response, as evidenced by the lack of goblet cell hyperplasia and decreased numbers of cells infiltrating the lungs (Fig. 2A). On the other hand, NPRC-/- mice that lack the ANP clearance receptor, NPRC, showed pathological effects similar to WT. Bronchoalveolar lavage (BAL) fluid from NPRA^-/- ^mice had reduced levels of the inflammatory cytokines, IL-4, IL-5 and IL-6, relative to wild type (Fig. 2B). From this result we reasoned that inhibition of ANP-NPRA signaling by siRNA against NPRA might be protective against airway inflammation and asthma.

### Selection of synthetic siNPRAs effective against NPRA

To test if transdermal siRNA nanoparticles can attenuate lung inflammation and asthma, we constructed three vector-driven siNPRAs targeting different regions of the human NPRA coding sequence. Inhibition of NPRA expression by different constructs was measured by Western blot assay. Based on their knockdown efficiency, we synthesized two siNPRA primers with sense strand sequence GGGCGCUGCUGCUGCUACCdTdT, and antisense strand GGUAGCAGCAGCAGCGCCCdTdT. Functional siNPRA was obtained by annealing the primers. As a control, a scrambled siRNA was also prepared. When synthetic siNPRA (0.1 nmol) was transfected into HEK-GCA cells, NPRA expression was significantly reduced compared to untreated controls or cells treated with scrambled siRNA (data not shown).

### Treatment with siNPRA and imiquimod cream decreased airway hyperresponsiveness

**Figure 3:**
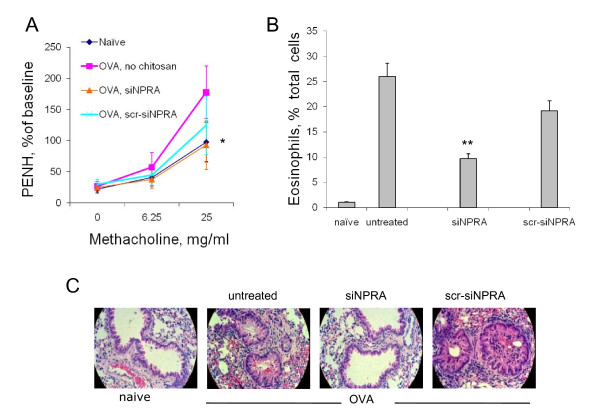
**Treatment with siNPRA and imiquimod cream reduced allergic airway hyperreactivity, lung eosinophilia and pathology**. (**A**) Transdermally-delivered siNPRA reduces airway hyperreactivity. Mice were sensitized to OVA, given the indicated treatments and challenged with OVA intranasally. AHR to methacholine challenge was recorded 24 h later in a whole-body plethysmograph which measures the enhanced pause (PENH). The PENH values for each methacholine concentration were averaged and expressed as a percentage of the PBS baseline reading. Results shown are averages of two separate experiments with standard deviations (*, *P *< 0.05). (**B**) Decrease in eosinophil numbers by siNPRA-imiquimod treatment. BAL cells were air dried and stained with a modified Wright's stain. Total cell numbers were approximately the same in each group and the number of eosinophils is given as percentage of the total. Treatment by siNPRA-imiquimod cream significantly reduced eosinophils in the BAL compared to controls Results shown are averages of two separate experiments with standard deviations (**, *P *< 0.01). (**C**) Reduction of lung inflammation by siNPRA-imiquimod cream. Lungs were removed, fixed in formalin and sectioned. Slides were stained with hematoxylin and eosin. Treatment with siNPRA caused a substantial decrease in lung inflammation, goblet cell hyperplasia and infiltration of inflammatory cells compared to the OVA control group and the group treated with scrambled siNPRA (scr-siNPRA) nanoparticles. Lung sections from naïve animals without any treatment show normal healthy lungs.

Topical treatment with siNPRA nanoparticle in imiquimod cream was tested to determine if it could attenuate airway inflammation. Several clinical parameters of asthma and biological markers of airway inflammation were evaluated. Four groups (4 mice per group, back hair shaved) of BALB/c mice were tested. The first group served as naive control while the second group received OVA sensitization and OVA challenge as the positive control. Animals in the third group got siNPRA treatment as well as OVA sensitization/challenge, while the last group was OVA-sensitized and – challenged, but treated with scrambled siNPRA nanoparticles. Airway hyperresponsiveness (AHR) to aerosol methacholine challenge (6.25 to 25 mg/ml) was measured 24 hrs after the final OVA challenge. It was found that the siNPRA-treated mice had significantly lower AHR than the OVA-positive control group or the group receiving scrambled siNPRA (Fig. 3A).

### Treatment with siNPRA and imiquimod cream reduced eosinophilia and lung pathology

The most direct indicator of airway inflammation is lung histopathology. For the purpose of measuring the number of eosinophils from animals of each group, BAL fluids were collected and BAL cells were fixed on slides by cytocentrifugation and stained using a differential cell staining kit. Eosinophils were counted microscopically and expressed as percentage of total cells. Fig. 3B shows the average eosinophil percentages from the four groups with different treatments. It is obvious that topical treatment with siNPRA nanoparticles mixed with imiquimod cream reduced eosinophil recruitment in the lung in this group. After H & E staining, lung sections from mice treated with siNPRA and imiquimod cream showed a substantial decrease in lung inflammation, goblet cell hyperplasia and infiltration of inflammatory cells compared to the untreated OVA group and the group treated with scrambled siRNA (Fig. 3C).

### Treatment with siNPRA and imiquimod cream reduced IL-4 and IL-5 levels

**Figure 4:**
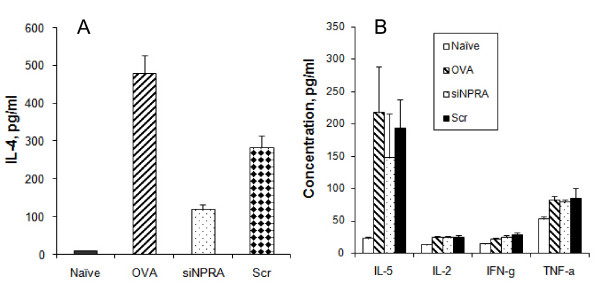
**Cytokine production in BALB/c mice is altered by siNPRA-imiquimod treatment**. (**A**) IL-4 in BAL fluid was measured by IL-4 ELISA. Significant reduction of IL-4 was achieved by siNPRA-imiquimod treatment compared to OVA controls. (**B**) Lungs of all animals from the four groups were removed and homogenized. The levels of IL-2, IL-5, IFN-γ and TNFα in lung homogenates were measured using a mouse Th1/Th2 Cytokine CBA kit. IL-5 was significantly downregulated by siNPRA treatment. Results shown are averages of two separate experiments with standard deviations (*, *P *< 0.05, **, *P *< 0.01).

The pro-inflammatory cytokines IL-4 and IL-5 are biological markers of airway inflammation. The levels of IL-4 and IL-5 were measured by ELISA or mouse Th1/Th2 Cytokine CBA kit following the manufacturer's instruction (BD Bioscience, CA). Significant reduction of IL-4 was observed in the siNPRA-treated group (Fig. 4A). IL-5 was also downregulated by siNPRA treatment (Fig. 4B). However, there was no significant change in IL-2, INF-γ and TNF-α when mice were treated with siNPRA nanoparticles compared to the untreated group or scrambled siRNA-treated group (Fig. 4B). Taken together, the observed changes in inflammatory cytokines, AHR and lung pathology demonstrate that siNPRA chitosan nanoparticles delivered through imiquimod cream can afford significant protection from airway allergy and inflammation.

## Discussion

Here we report that a topical cream containing siNPRA and imiquimod modulates lung inflammation in a mouse model of allergic asthma. Both imiquimod and siNPRA showed anti-inflammatory effect in our test. However, siNPRA was the dominant protective agent as evidenced by comparison with the relatively low reduction in inflammation in the scrambled siRNA-treated group in which the protection resulted from imiquimod alone. However, besides the anti-inflammatory effect of imiquimod, the penetrating agent in the imiquimod cream also facilitated the penetration siNPRA. To the best of our knowledge, this is the first report of the transdermal delivery of synthetic siRNA.

Transdermal delivery of biomolecules and drugs has several advantages over other delivery routes. First, it is painless and therefore a boon to patients who require frequent drug administration. Second, the cream is simple to apply and particularly useful for treating asthmatic infants who cannot be given drugs easily or safely by oral, intranasal or inhalational routes. A transdermal cream to administer the chitosan-conjugated nanocomplexes is expected to be safe and effective and may have advantages over electroporation or particle-mediated epidermal delivery of DNA/RNA in which transient skin irritation was observed [24,25]. The capability of biocompatible chitosan nanoparticles for transdermal delivery of siRNA makes chitosan a very promising agent for treating asthma and other diseases especially in children. The nanocomplexation with chitosan may contribute to easier penetration of siRNA through the outermost barriers of the skin and may also provide longer duration of siRNA *in vivo*.

Our results suggest that the ANP-NPRA signaling pathway plays an important role in inflammation of the airway and that prevention and control of pathology could be achieved by inhibition of ANP signaling. We found that increased production of ANP induced airway inflammation in normal mice and augmented inflammation in a murine model of allergen-induced asthma. NPRA^-/- ^mice exhibit significantly lower inflammation of the lung compared to wild-type mice. This result is consistent with our previous finding that NP73-102, an inhibitor of NPRA, decreased several pro-inflammatory transcription factors in the lung [15]. Increased airway inflammation is associated with activation of the transcription factors nuclear factor-kappa B (NFκB) and activator protein-1 (AP1), and the extracellular signal-regulated receptor kinase (Erk1/2). ANP also reduces TNF-α-induced actin polymerization and endothelial permeability and increases cytoprotective proteins such as hemeoxygenase-1 [26]. In human lung epithelial cells, intracellular expression of ANP together with the synthetic natriuretic peptide, NP73-102, decreased activation of NFκB, AP-1 and Erk 1, 2. NP73-102 possesses anti-inflammatory activity and is capable of preventing pulmonary inflammation when given prophylactically or therapeutically. The evidence that NPRA-/- mice have less eosinophilia and lower levels of Th2-like cytokines compared to wild type indicate that the ANP pathway is pro-Th2, and this is consistent with a previous study which showed that human DCs exposed to ANP promoted TH2-like cytokine expression.

Transdermally delivered siNPRA significantly decreased lung inflammation in BALB/c mice as evident from lung section staining, eosinophil counting and quantitation of Th2-like cytokines IL-4 and IL-5. These results are in agreement with the previous reports that activation of the ANP pathway increases Th2 dominance. Also, siNPRA-treated BALB/c mice exhibit significantly lower airway hyperresponsiveness than those receiving scrambled siRNA. This indicated that in addition to its anti-inflammatory activity, knockdown of NPRA by siNPRA also attenuates AHR which operates through a different set of genes from the inflammatory cytokines.

In summary, we demonstrate that synthetic chitosan-siRNA nanocomplexes can be effectively delivered transdermally. The lack of pulmonary inflammation in mice deficient in NPRA or in mice treated by siNPRA provides compelling evidence for the role of ANP-NPRA signaling in pulmonary inflammation. Moreover, transdermally applied siNPRA chitosan nanoparticles have proven safe and effective in mice and may provide an innovative new treatment approach for preventing airway inflammation and asthma in humans.

## Conflict of interests

The author(s) declare that they have no competing interests.

## Authors' contributions

XW and XK performed the studies presented in Figures 2, 3, 4. WX and SM contributed to the data shown in Figure 1. XL, RFL collaborated on the project. SSM conceived and designed the experiments. All authors have read and approved the manuscript.
